# Intertwined regulation between RNA m^6^A modification and cancer metabolism

**DOI:** 10.1016/j.cellin.2022.100075

**Published:** 2022-12-05

**Authors:** Jiaxu Liu, Hao Huang, Minghao Zhang, Guoliang Qing, Hudan Liu

**Affiliations:** a Department of Hematology, Medical Research Institute, Zhongnan Hospital of Wuhan University, Wuhan University, Wuhan, China; b Frontier Science Center of Immunology and Metabolism, Wuhan University, Wuhan, China

## Abstract

RNA N6-methyladenosine (m^6^A) has been identified as the most common, abundant and conserved internal modification in RNA transcripts, especially within eukaryotic messenger RNAs (mRNAs). Accumulating evidence demonstrates that RNA m^6^A modification exploits a wide range of regulatory mechanisms to control gene expression in pathophysiological processes including cancer. Metabolic reprogramming has been widely recognized as a hallmark of cancer. Cancer cells obtain metabolic adaptation through a variety of endogenous and exogenous signaling pathways to promote cell growth and survival in the microenvironment with limited nutrient supply. Recent emerging evidence reveals reciprocal regulation between the m^6^A modification and disordered metabolic events in cancer cells, adding more complexity in the cellular network of metabolic rewiring. In this review, we summarize the most recent advances of how RNA methylation affects tumor metabolism and the feedback regulation of m^6^A modification by metabolic intermediates. We aim to highlight the important connection between RNA m^6^A modification and cancer metabolism, and expect that studise of RNA m^6^A and metabolic reprogramming will lead to greater understanding of cancer pathology.

## 1 Introduction

Generations of RNA transcripts are crucial molecular events to convey the genetic information (DNA sequences) to active proteins or functional RNA if intrinsically non-coding. There are more than 150 distinct chemical modifications on cellular RNA that have been identified to date. *N*-methyladenosine (m^6^A) is the most abundant and the best-characterized RNA modification in eukaryotic cells (Zaccara et al., 2019). Emerging evidence suggests that m^6^A is involved in a variety of RNA metabolic events including pre-mRNA splicing, 3′-end processing, nuclear export, translation regulation and mRNA decay (Gilbert et al., 2016; Roignant & Soller, 2017). As such, this RNA modification provides an extra layer of intricate regulation in gene expression. Similar to DNA and histone methylation, m^6^A is also dynamic and reversible in mammalian cells and considered as additional epigenetic regulation to affect fundamental cellular processes including proliferation and survival. Numerous studies have shown that m^6^A RNA methylation is widely involved in regulation of immune responses (Shulman & Stern-Ginossar, 2020), embryonic development (Song et al., 2021), brain development (Meyer et al., 2012), as well as human diseases as exemplified by cancer (Pan et al., 2018; Sun et al., 2019).

Metabolic reprogramming is one of the most prominent hallmarks of cancer (Hanahan & Weinberg, 2011). The common feature of cancer cell metabolism is the ability to acquire essential biomacromolecules from environment and utilize these nutrients to maintain viability and build new biomass. In order to fulfill the biosynthetic demands associated with proliferation, cancer cells have evolved specific metabolic adaptations (Dong et al., 2020). Tumor cells typically exhibit increased aerobic glycolysis, despite available oxygen, by taking up glucose avidly and converting a majority of glucose-derived pyruvate to lactate, a phenomenon known as the Warburg effect. Because of this, cancer cells depend on glutamine anaplerosis to replenish the tricarboxylic acid (TCA) cycle intermediates for macromolecular biosynthesis and nicotinamide adenine dinucleotide phosphate (NADP) production (Gao et al., 2009; Wise et al., 2008; Yuneva et al., 2007). In addition, malignant cells must also acquire metabolic changes of lipids and nucleic acids. Enhanced synthesis or uptake of lipids contributes to rapid cancer cell growth and tumor formation (Cheng et al., 2018). Moreover, tumor cells show an altered nucleotide metabolism compared with normal cells, as manifested by higher activity of the nucleotide anabolic pathway as well as lower activity of the nucleotide catabolic pathway (Lane & Fan, 2015). All of these alterations together support cellular biomass synthesis and energy storage, rendering pro-survival adaptive responses of tumor cells to a variety of stressed conditions (Carracedo et al., 2013; Lane & Fan, 2015).

In this review, we discuss recent findings that how m^6^A modification affects metabolic pathways and tumor progression, and how metabolic intermediates in turn regulate RNA N6-methylation. These findings have reshaped our insights in RNA m^6^A-mediated epigenetic regulation and highlight the importance of intertwined link between m^6^A and cancer metabolism. We expect the studies of m^6^A and metabolic reprogramming to foster a deeper understanding of pathological mechanisms in cancer and unlock new therapeutic opportunities.

## 2 RNA m^6^A modification

Methylation of adenosine at the N6 position is the most abundant internal mRNA modification in eukaryotic species (Zaccara et al., 2019). The presence of m^6^A was first discovered on mammalian mRNA in the early 1970s (Desrosiers et al., 1974). The rapid development of next-generation sequencing (NGS) technology has advanced our understanding of the landscape of m^6^A in the transcriptome, and revealed a previously hidden plethora of m^6^A modifications. They are not only present in protein-coding mRNA but also in non-coding RNAs including long noncoding RNA (lncRNA) (Patil et al., 2016; Zhou et al., 2016), ribosomal RNA (rRNA) (Ma et al., 2019; van Tran et al., 2019), and small nuclear RNA (snRNA) (Aoyama et al., 2020; Mauer et al., 2019).

Mounting evidence manifest the widespread prevalence of m^6^A in mRNA. Most m^6^A-modified mRNAs bear only a single m^6^A site, but some mRNAs contain 20 or more m^6^A sites (Dominissini et al., 2012; Linder et al., 2015; Meyer et al., 2012). Previous studies of the correlation of m^6^A abundance with the structure of specific transcripts indicate that long internal exons (much larger than ∼140 bp) were considered strong inducers of m^6^A addition to transcribed mRNAs (Batista et al., 2014; Dominissini et al., 2012; Geula et al., 2015; Ke et al., 2015; Ke et al., 2017). Recent transcriptome-wide m^6^A locus mapping reveals its distribution across thousands of transcripts. The m^6^A modification sites have a typical consensus sequence DRACH (D = G, A or U; R = G or A; H = A, C, or U), and are enriched in the coding sequence (CDS) and 3′ untranslated region (3′UTR), with a particularly high enrichment around the stop codon (Dominissini et al., 2012; Linder et al., 2015). Analysis of mRNAs with a large number of m^6^A sites shows that the m^6^A modification is more selectively enriched in a subset of mRNAs such as genes regulating development and cell fate specification (Linder et al., 2015; Schwartz et al., 2014), whereas transcripts encoding “housekeeping” genes had little m^6^A enrichment, such as ribosomal proteins (Dominissini et al., 2012; Geula et al., 2015; Meyer et al., 2012; Schwartz et al., 2014).

## 3 Dynamic regulation of RNA m^6^A modification

This dynamic and reversible m^6^A modification process can be coregulated by three factors called “writer”, “eraser” and “reader” (Meyer & Jaffrey, 2017; Zaccara et al., 2019). The m^6^A in mRNAs and other RNA polymerase II derived transcripts are primarily methylated by the canonical complex of writers comprised of methyltransferase-like 3 (METTL3), methyltransferase-like 14 (METTL14) and wilms tumor 1-associated protein (WTAP) (Ping et al., 2014). METTL3, an *S*-adenosylmethionine (SAM)-binding protein, is the catalytic core with enzymatic functions. It is interesting to note that recent findings in gastric cancer also suggest a methyltransferase-independent role of METTL3 in malignant cells (Wei et al., 2022). METTL14 is another essential active component that stabilizes METTL3 and facilitates its binding to RNA. They form a stable core heterodimeric complex. WTAP lacks a conserved methylation domain and has no catalytic activity by itself; it binds to METTL3/14 complex as an adaptor protein and is required for optimal substrate recruitment and METTL3/14 localization (Zaccara et al., 2019).

The FTO (fat mass and obesity associated protein) and ALKBH5 (alpha-ketoglutarate-dependent dioxygenase AlkB homolog 5) are two well-characterized m^6^A demethylases (also termed m^6^A erasers). Both proteins belong to the family of α-ketoglutarate (α-KG)-dependent dioxygenases that catalyze m^6^A demethylation in a Fe^2+^ and α-KG-dependent manner (Jia et al., 2011; Zheng et al., 2013). FTO is the first confirmed m^6^A demethylase, which also catalyzes the demethylation of m^6^A_m_ on mRNA and m^1^A on tRNA (Jia et al., 2011; Linder et al., 2015; Wei et al., 2018). It is generally believed that internal m^6^A modifications in mRNAs are the major substrates of FTO in many cell types studied so far, including acute myeloid leukemia (AML) cells and melanoma cells (Li et al., 2017; Su et al., 2018; Wei et al., 2018; Yang et al., 2019; Zhang et al., 2019). In contrast, ALKBH5 seems to be an m^6^A-specific demethylase, which has no activity towards m^6^A_m_ (Mauer et al., 2017). Since ALKBH5 locates in the nucleus and appears to be concentrated in nuclear speckles (Zheng et al., 2013), ALKBH5 most likely demethylates m^6^A in nuclear RNA during its biogenesis in the nucleus. ALKBH5 and FTO have demonstrated oncogenic roles in a variety of malignancies, due to regulation of distinct population of downstream targets. Importantly, these “writer” and “eraser” proteins determine the dynamic and reversible regulation of m^6^A RNA modification and consequently gene expression, allowing instant adaptation of tumor cells in response to microenvironment change.

The m^6^A modifications are recognized by specific proteins (also termed “reader”) to determine the RNA fates in various biological contexts. Studies using methylated RNA probes to pull down binding proteins followed by mass spectrometry analysis have identified several m^6^A reader candidates in mammalian cells (Dominissini et al., 2012). Some of these reader proteins, including YTHDC1 (DC1), YTHDC2 (DC2), YTHDF1 (DF1), YTHDF2 (DF2) and YTHDF3 (DF3), contain a YTH (YT521B homology) domain. Another category of reader proteins are FMRP and IGF2BP proteins, which bind a common sequence motif DR (m^6^A)CH (Edupuganti et al., 2017; Huang et al., 2018). These m^6^A readers can recruit diverse regulatory or functional machinery to m^6^A-containing RNA, and impact the fate of target RNA including alterations of RNA stability (Wang et al., 2014a, 2014b), translation efficiency (Meyer et al., 2015; Wang et al., 2015), and splicing (Xiao et al., 2016).

## 4 RNA m^6^A regulates tumor metabolism

**Fig. 1:**
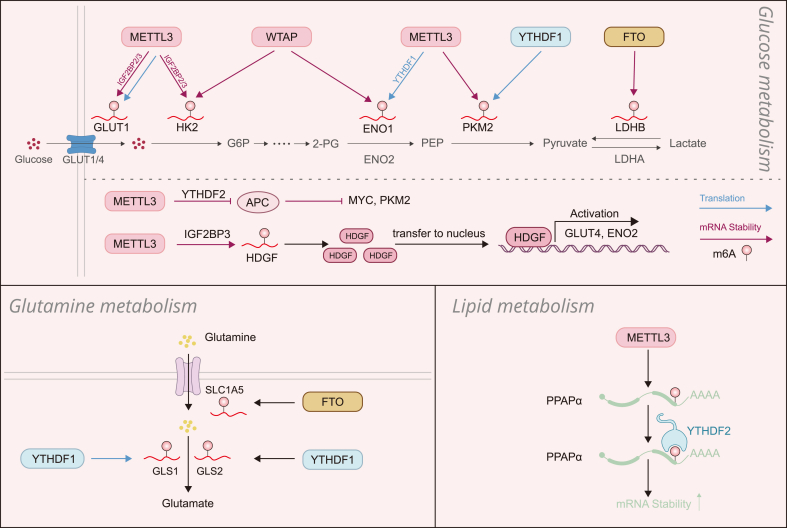
**The RNA m**^**6**^**A methylation of key metabolic enzymes or regulators modulates glucose, glutamine and lipid metabolism.** In glucose metabolism, The METTL3/WTAP complex catalyzes the m^6^A modification in transcripts of *GLUT1*, *HK2*, *ENO1* and *PKM2* and promotes the mRNA stability or enhances the translation. The m^6^A reader YTHDF1 accelerates the translation of *PKM2* and the m^6^A demethylase FTO promotes the mRNA stability of *LDHB*. METTL3 also indirectly affects metabolic enzyme expression via adding m^6^A on the *APC* and *HDGF* transcripts. In glutamine metabolism, FTO and YTHDF1 promote the mRNA stability of *SLC1A5* and *GLS2* respectively. YTHDF1 enhances the *GLS1* translation. In lipid metabolism, METTL3 and YTHDF2 coordinate to promote the *PPAPα* mRNA stability. See the text for more details.

Cancer cell metabolism is geared toward biomass synthesis and cell proliferation. To maintain growth in the condition where finite nutrient resources may be inadequate for rapid tumor growth, cancer cells employ a variety of adaptions in metabolic pathways that control glucose, glutamine and lipid metabolism (Hanahan & Weinberg, 2011). Emerging evidence suggests that m^6^A-containing genes were significantly enriched in cancer metabolism-related pathways (Han et al., 2021), indicating that a vital role of RNA m^6^A in regulation of metabolic changes. We here summarize the latest findings of how m^6^A RNA methylation coordinates metabolic reprogramming for tumor progression (Fig. 1).

### 4.1 RNA m^6^A regulates glucose metabolism

Metabolism of glucose, the major nutrient resource, mainly includes glycolysis pathway in cytoplasm and TCA cycle in mitochondria. Glycolysis is a central pathway of glucose metabolism and the metabolite pyruvate can be converted into lactate as the end product for extracellular secretion normally during hypoxia, or enter into the mitochondria for TCA cycle and oxidative phosphorylation in normoxia. Tumor cells, unlike normal cells, depend largely on glycolysis for producing energy even in the presence of adequate levels of oxygen (Dong et al., 2020).

High-throughput MeRIP sequencing in colorectal cancer (CRC) suggests that the m^6^A associated genes are enriched in glucose metabolic pathways, highlighting the crucial role of m^6^A modifications in regulating glucose metabolism (Zhang et al., 2021). METTL3-mediated m^6^A modification in the glucose transporter protein type 1 (GLUT1, also named SLC2A1) enhances its mRNA translation, which promotes glucose uptake and subsequently lactate production, leading to CRC progression (Chen et al., 2021). Another study has also demonstrated that METTL3 directly catalyzes m^6^A modifications of HK2 and GLUT1 mRNA, and these modified RNAs are recognized by the m^6^A reader YTHDF1 and/or IGF2BP2/3 and acquire increased RNA stability, promoting aerobic glycolysis (Liu et al., 2021; Shen et al., 2020; Wang, Guo, et al., 2020). Similar findings are shown in gastric cancer that WTAP enhances the stability of HK2 mRNA by facilitating m^6^A modifications in the 3′UTR (Yu et al., 2021). Increased enolase 1 (ENO1) mRNA m^6^A modification facilitates its binding to the m^6^A reader YTHDF1 and results in enhanced translation, leading to aerobic glycolysis in human lung adenocarcinoma and breast cancer (Ma et al., 2022; Ou et al., 2021). Paradoxically, aberrant expression of the demethylase FTO has also been found in hepatocellular carcinoma (HCC) and predicts poor prognosis. As a dependency factor in HCC, FTO triggers the demethylation of pyruvate kinase M2 (PKM2) mRNA, resulting in enhanced mRNA translation (Li et al., 2019). Likewise, the m^6^A reader YTHDF1 enhances glycolysis in breast cancer cells by upregulating PKM2 (Yao et al., 2022). FTO has recently been reported upregulating phosphofructokinase platelets (PFKP) and lactate dehydrogenase B (LDHB) via demethylase activity in AML (Qing et al., 2021). It appears that the tumor dependencies on m^6^A writers, readers and erases are context specific. The mechanism underlying the dependent selectivity remains unresolved, most likely attributable to unique target RNAs that encode vital tumorigenic factors or which category of m^6^A reader proteins bind to RNAs in respective circumstance.

Many “star” transcription factors, such as MYC and HIF1α, govern tumor metabolic reprogramming, and expression of these master regulators can also be modulated by m^6^A methylation. Suppressed expression of FTO in lung adenocarcinoma significantly enhanced m^6^A levels in MYC mRNA, thereby promoting MYC protein expression and subsequent increase in glycolysis and tumor cell proliferation (Yang, Shao, et al., 2021). In oral squamous cell carcinoma (OSCC), METTL3 targets the 3′UTR (near the stop codon) of the MYC transcript to install m^6^A modifications that are recognized by YTHDF1 to enhance its stability (Zhao et al., 2020). Interestingly, inhibition of FTO in AML increases m^6^A RNA modification in cells, which in turn reduces the stability of MYC transcripts, leading to suppression of AML (Su et al., 2018). Thus, mRNA m^6^A modification results in opposite outcomes of MYC RNA transcripts, stabilization or degradation, in different tumor types, probably due to distinct reader proteins that induce variable consequences. The m^6^A modification of HIF1α mRNA, according to existing studies, consistently promotes RNA stability in multiple human cancers (Jiang et al., 2021; Shmakova et al., 2021; Yang, Wang, et al., 2021), and consequently enhances the glycolysis pathway.

Apart from the direct regulation of metabolic enzymes and metabolically related transcription factors, there exist also indirect mechanisms. METTL3 upregulates the m^6^A modification of *adenomatous polyposis coli* (*APC*) RNA in esophageal squamous cell carcinoma (ESCC) cells, which recruits YTHDF for *APC* mRNA degradation. Reduced APC expression unleashes the suppression of MYC and PKM2, thereby leading to enhanced aerobic glycolysis, tumor cell proliferation and tumor formation (Wang et al., 2021). In gastric cancer cells, METTL3 stimulates the m^6^A modification of heparin binding growth factor (HDGF) mRNA, and IGF2BP3 then directly binds to the m^6^A-modified mRNA and enhanced RNA stability. HDGF can translocate into nucleus and activate the transcription of glucose transporter *GLUT4* and the glycolytic enzyme *ENO2*, resulting in enhanced glycolysis and subsequent tumor growth and liver metastasis (Wang, Chen, et al., 2020). These findings support the notion that regulation of glycolysis by the m^6^A modification of key factors has a significant impact on the onset and development of human cancers.

### 4.2 RNA m^6^A regulates glutamine metabolism

Glutamine supplies a major source of carbon and nitrogen to support the biosynthesis, energetics and cell homeostasis that cancer cells may use to promote tumor growth (Altman et al., 2016). Glutamine can be transported into cells through transmembrane transporters (Bhutia et al., 2015), such as SLC1A5 (also known as ASCT2) (Nicklin et al., 2009; Wise & Thompson, 2010). In clear cell renal cell carcinoma (ccRCC), comprehensive analysis of m^6^A-seq and mRNA-seq analysis identified the glutamine transporter SLC1A5 as an FTO target, and the FTO-SLC1A5 axis is crucial for the metabolic reprogramming and survival of ccRCC cells (Xiao et al., 2020).

Glutaminase isoenzymes (GLS1 and GLS2) are the key enzymes for glutamine catabolism, also termed glutaminolysis. In mitochondria, GLS1 or GLS2 catalyzes glutamine to glutamate, and glutamate is then converted into α-ketoglutarate, which enters the tricarboxylic acid cycle for continuing catabolism (Masisi et al., 2020). Previous reports show that GLS2 is the downstream target of METTL3, which promotes GLS2 expression. Their findings identify a METTL3-GLS2 signaling as a potential therapeutic target in antimetastatic strategies against ESCC (Chen, Huang, et al., 2021). Similar findings suggest that GLS1 and YTHDF1 are significantly up-regulated in cisplatin-resistant CRC cells. GLS1 mRNA 3′UTR bears m^6^A modifications and contains a putative YTHDF1 binding motif, enhancing GLS1 protein synthesis efficiency and thereby conferring cisplatin resistance through increased glutamine metabolism (Chen, Liu, et al., 2021).

### 4.3 RNA m^6^A regulates lipid metabolism

Mammalian cells obtain lipids through two mechanisms, *de novo* synthesis and uptake from outside. Increased lipid uptake, storage, and lipogenesis occur in a variety of cancers and contribute to the rapid growth of tumors (Cheng et al., 2018). Peroxisome proliferator-activated receptor-α (PPARα), PPARδ and PPARγ play critical physiological roles as lipid sensors and regulators of lipid metabolism. They are transcription factors that regulate gene expression following activation by ligands such as fatty acids (Montaigne et al., 2021). A recent interesting finding suggests mRNA m^6^A impacts circadian regulation of lipid metabolism. Deletion of Bmal1 from the liver shifts mRNA methylation patterns, and particularly increases m^6^A levels of PPARα mRNA, resulting in increased lipid accumulation in cells. These data show that RNA m^6^A is important for circadian regulation of downstream genes and lipid metabolism (Zhong et al., 2018).

Above all, it is interesting to note that both methylases and demethylases can regulate metabolic pathways and show pro-tumorigenic roles. This is very likely resulting from distinct downstream effectors in various tumor contexts. The precise mechanism of m^6^A modification in regulation of cancer metabolism remains largely unexplored, and awaits more attention and further investigation.

## 5 Metabolites participating in RNA m^6^A regulation

**Fig. 2:**
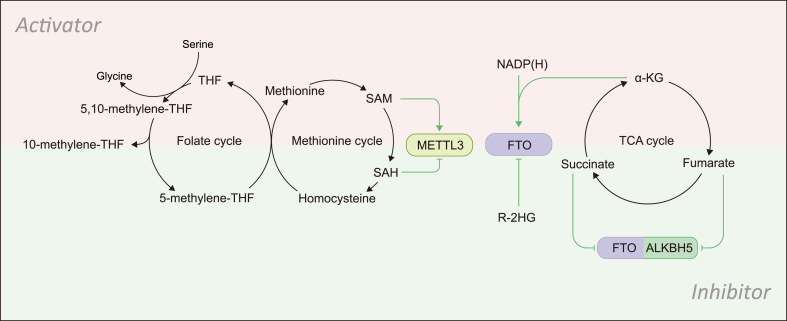
**Metabolite intermediates modulate the key components of the m**^**6**^**A machinery.** The abundance of methyl group donor SAM, produced from one-carbon metabolism, controls the methyl transferase reaction catalyzed by METTL3. The ALKBH5 and FTO demethylase activity can be modulated by NADP(H), α-KG, R-2HG, succinate and fumarate. See the text for more details.

While metabolic pathways are modulated by m^6^A modification of RNA transcripts encoding metabolic enzymes or regulators, some metabolites also affect cellular m^6^A level via interaction with the m^6^A machinery (Fig. 2).

### 5.1 SAM and SAH

Like other typical enzymatic chemical reaction, the writer-mediated mRNA methylation is regulated by the abundance of products and substrates. SAM is the universal methyl donor for the cellular methylation process, and serves as an important substrate for m^6^A modification. METTL3 is known as a SAM-binding protein that exhibits enzymatic activity (Bokar et al., 1997). In the catalytic reaction of adenosine methylation, SAM transfers a methyl group to adenosine on RNAs and converts to *S*-adenosyl homocysteine (SAH). SAM is produced by the one-carbon metabolism including folate and methionine cycles. In this metabolic pathway, serine and methionine are carbon donors that serine is converted to glycine, providing a one-carbon unit to tetrahydrofolate (THF). Subsequently, homocysteine receives one-carbon from methyl-THF and becomes methionine which provides methyl group and produce SAM (Locasale, 2013). Deregulation of these cycles may reduce SAM synthesis and impair the cellular m^6^A modification resulting in altered gene expression (Villa et al., 2021). Alternatively, METTL3 methyltransferase activity can be inhibited by SAH, the product of this methylation reaction that acts as a strong allosteric inhibitor (Li et al., 2016). In addition, the folate binding protein glycine *N*-methyltransferase (GNMT) catalyzes the reaction transferring a methyl group from SAM to glycine generating SAH and *N*-methylglycine therefore decreasing SAM accumulation and altering the SAM/SAH ratio (; Kerr, 1972). As such, GNMT has been suggested as a tumor suppressor in HCC by hampering enzymic activity of METTL3 (Hughey et al., 2018). In sum, the SAM/SAH ratio is capable of regulating m^6^A modification by modulating the METTL3-mediated enzymatic reaction.

### 5.2 α-KG and R-2-hydroxyglutarate (R-2HG)

α-KG, also known as 2OG or 2-oxoglutarate, one of the key metabolic intermediates in TCA cycle, is essential for FTO and ALKBH5 activity. As FTO and ALKBH5 are both α-KG-dependent dioxygenase family protein, α-KG, oxygen (O_2_) and Fe(II) (non-heme iron) are required to constitute complete enzymatic activity (Losman et al., 2020; Xu & Bochtler, 2020). As such, fluctuation of cellular α-KG abundance indirectly affects the m^6^A level through regulating the enzymatic activity of FTO and ALKBH5. Isocitrate dehydrogenase 1 and 2 (IDH1/2) catalyze the oxidative decarboxylation of isocitrate to α-KG in an NADP^+^-dependent manner in the TCA cycle. Recurrent somatic mutations in IDH1 and IDH2 occur in 80% of grade II-III gliomas and secondary glioblastoma, 10%–20% of AML patients. Mutant IDH1 and 2 acquire the ability to convert α-KG to R-2HG (Dang et al., 2009). As R-2HG has the similar structure compared with α-KG, it can competitively bind to the α-KG-dependent dioxygenase particularly FTO, and restrain the demethylase activity of FTO (Xu et al., 2011), resulting in increased cellular m^6^A level. Since IDH mutants block cell differentiation and promote tumor transformation, and inhibition of mutant IDH (IDHi) can reverse this effect, R-2HG (as the major metabolic product of IDH mutants) has been regarded as an oncometabolite (Rohle et al., 2013; Wang et al., 2013). A recent intriguing finding shows that R-2HG also displays a broad and intrinsic anti-tumor activity in leukemia and glioma by targeting FTO. R-2HG increases global m^6^A RNA modification, thereby induces suppression of MYC/CEBPA-associated pathways and aerobic glycolysis through affecting mRNA m^6^A modification and the fates of target RNAs (Qing et al., 2021; Su et al., 2018).

### 5.3 Citrate, succinate and fumarate

In addition to α-KG, biochemical evidence suggests that other metabolites in citric acid cycle can also impact the enzymatic activity of demethylases ALKBH5 and FTO. For instance, citrate was found to occupy the α-KG-binding pocket of ALKBH5 and FTO (Aik et al., 2013; Feng et al., 2014), thereby repressing the enzymatic activity. Succinate and fumarate are the downstream products of α-KG which exhibit similar molecular structures to α-KG as well. These metabolic intermediates can act as binding competitors toward FTO and ALKBH5 thus inhibit m^6^A demethylases. As these findings are all based on a cell-free system (Aik et al., 2013; Feng et al., 2014), whether the mitochondria-compartmentalized TCA cycle metabolites regulate cellular m^6^A demethylation awaits further validation. Most likely, citrate, succinate and fumarate would affect global m^6^A modification in cells and yield R-2HG-like phenotypic consequences.

### 5.4 NADP(H)

The nicotinamide adenine dinucleotide (NAD+)/reduced NAD+ (NADH) and NADP+/reduced NADP+ (NADPH) redox couples are essential for maintaining cellular redox homeostasis. They are also required for cellular metabolism and reductive biosynthesis. Deficiency or imbalance of these two redox couples alters cellular redox and metabolic hemostasis (Xiao et al., 2018). Recent report shows an exciting finding that NADH and NADPH can potentiate FTO activity (Wang, Song, et al., 2020). Based on a fluorescence screen of metabolites that bind to FTO, NADH and NADPH were found to be the strongest binding partners and activators of FTO. These results were further validated *in vivo*, suggesting that the reducing potential of NADPH and NADH may be used for demethylation reactions. Intriguingly, these molecules were not consumed by FTO, and the concentration remained constant during demethylation reactions. Further mechanistic studies are expected to better understand the molecular mechanisms of NADPH-dependent activation of m^6^A demethylases as well as the biological significance.

## 6 Concluding remarks

Increasing evidence has demonstrated the indispensable role of m^6^A modification in tumor metabolism by directly regulating the expression of metabolic enzymes, signaling molecules or transcription factors governing metabolic pathways. RNA m^6^A modification manifests a prominent mechanism in regulating a wide range of metabolic events including glucose, glutamine and lipid catabolism. Conversely, a variety of metabolites in cancer cells can also interact with the m^6^A machinery and modulate the RNA methylation pattern, thereby altering gene expression to facilitate neoplastic transformation. More evidence will be reported to support the intertwined link between RNA m^6^A modification and metabolic alterations. Further investigations, particularly *in vivo*, are expected to consolidate the tumorigenic role of these reciprocal regulations under pathological state. On the basis of these findings, small molecule drugs that target the m^6^A machinery will induce metabolic defects and vice versa. Therefore, simultaneous targeting of RNA m^6^A modifications and metabolic pathways may yield synergistic anti-tumor effects, paving a new way for cancer treatment modality.

## Abbreviation

3′UTR: 3′ untranslated region
AML: acute myeloid leukemia
APC: adenomatous polyposis coli
ALKBH5: alpha-ketoglutarate-dependent dioxygenase AlkB homolog 5
ccRCC: clear cell renal cell carcinoma
CDS: coding sequence
CRC: colorectal cancer
ENO1: enolase 1
ESCC: esophageal squamous cell carcinoma
FTO: fat mass and obesity associated protein
GLUT1: glucose transporter protein type 1
GLUT4: glucose transporter protein type 4
GLS1/2: glutaminase isoenzymes 1 and 2
GNMT: glycine *N*-methyltransferase
HDGF: heparin binding growth factor
HCC: hepatocellular carcinoma
LDHB: lactate dehydrogenase B
lncRNA: long noncoding RNA
mRNAs: messenger RNAs
METTL14: methyltransferase-like 14
METTL3: methyltransferase-like 3
NGS: next-generation sequencing
NAD: nicotinamide adenine dinucleotide
NADP: nicotinamide adenine dinucleotide phosphate
OSCC: oral squamous cell carcinoma
PPARα: peroxisome proliferator-activated receptor- α
PFKP: phosphofructokinase platelets
PKM2: pyruvate kinase M2
R-2HG: R-2-hydroxyglutarate
rRNA: ribosomal RNA
m^6^A: RNA N6-methyladenosine
SAH: *S*-adenosyl homocysteine
SAM: *S*-adenosylmethionine
snRNA: small nuclear RNA
IDH1/2: socitrate dehydrogenase 1 and 2
SLC1A5: solute carrier family 1 member 5
THF: tetrahydrofolate
TCA: tricarboxylic acid
WTAP: wilms tumor 1-associated protein
α-KG: α-ketoglutarate
